# GRASPx: efficient homolog-search of short peptide metagenome database through simultaneous alignment and assembly

**DOI:** 10.1186/s12859-016-1119-1

**Published:** 2016-08-31

**Authors:** Cuncong Zhong, Youngik Yang, Shibu Yooseph

**Affiliations:** Informatics Department, J. Craig Venter Institute, La Jolla, CA 92037 USA

## Abstract

**Background:**

Metagenomics is a cultivation-independent approach that enables the study of the genomic composition of microbes present in an environment. Metagenomic samples are routinely sequenced using next-generation sequencing technologies that generate short nucleotide reads. Proteins identified from these reads are mostly of partial length. On the other hand, *de novo* assembly of a large metagenomic dataset is computationally demanding and the assembled contigs are often fragmented, resulting in the identification of protein sequences that are also of partial length and incomplete. Annotation of an incomplete protein sequence often proceeds by identifying its homologs in a database of reference sequences. Identifying the homologs of incomplete sequences is a challenge and can result in substandard annotation of proteins from metagenomic datasets. To address this problem, we recently developed a homology detection algorithm named GRASP (Guided Reference-based Assembly of Short Peptides) that identifies the homologs of a given reference protein sequence in a database of short peptide metagenomic sequences. GRASP was developed to implement a simultaneous alignment and assembly algorithm for annotation of short peptides identified on metagenomic reads. The program achieves significantly improved recall rate at the cost of computational efficiency. In this article, we adopted three techniques to speed up the original version of GRASP, including the pre-construction of extension links, local assembly of individual seeds, and the implementation of query-level parallelism.

**Results:**

The resulting new program, GRASPx, achieves >30X speedup compared to its predecessor GRASP. At the same time, we show that the performance of GRASPx is consistent with that of GRASP, and that both of them significantly outperform other popular homology-search tools including the BLAST and FASTA suites. GRASPx was also applied to a human saliva metagenome dataset and shows superior performance for both recall and precision rates.

**Conclusions:**

In this article we present GRASPx, a fast and accurate homology-search program implementing a simultaneous alignment and assembly framework. GRASPx can be used for more comprehensive and accurate annotation of short peptides. GRASPx is freely available at http://graspx.sourceforge.net/.

**Electronic supplementary material:**

The online version of this article (doi:10.1186/s12859-016-1119-1) contains supplementary material, which is available to authorized users.

## Background

Metagenomics allows for a snapshot of the genomic content of all microbes within a specific environmental niche and is not limited by current microbial cultivation barriers. High-throughput shotgun sequencing is routinely applied on collected metagenomic samples, generating a large number of short DNA sequences (*reads*). A key analysis step is to infer the functions of the protein sequences predicted from these reads, hereafter referred to as *annotation*. The annotation problem is conceptually equivalent to the homology detection problem: given a reference sequence with known annotation, finding all homologous reads of the reference from the read set and subsequently transferring the annotation of the reference to the homologs. Intuitively, the problem can be solved using a direct alignment approach, simply aligning the reference against the individual reads (e.g. using BLAST [1], FASTA [2], RAPSearch [3, 4], and DIAMOND [5] etc.). An alternative *de novo* assembly approach assembles the individual reads into contigs (e.g. using SPAdes [6], Meta-Velvet [7], Meta-IDBA [8], and SOAPdenovo [9] etc.), which correspond to near-complete or complete protein sequences that are easier to annotate. Given the annotation of the contigs, the annotation of the reads can be inferred through their placement in the contigs. It has been shown that, for protein-sequence reconstruction, the assembly of short peptides (identified from DNA reads using MetaGeneAnnotator [10] or FragGeneScan [11] etc.) is more effective than the assembly of the nucleotide reads themselves [12, 13]. Either or both of the direct alignment and the *de novo* assembly approaches could be applied depending on specific applications and available computational resources.

Each of the direct alignment and the *de novo* assembly approaches has its own limitations. Gene calling and homology search with short reads are, in general, more challenging than with complete sequences. On the other hand, reconstructing full-length genome (assembly) is time consuming and can frequently ignore low-coverage organisms, therefore making the detection of low-abundant genes difficult and incomplete. To tackle these limitations, the annotation problem can be reformulated into the *gene-centric assembly* problem, which, given a reference protein of interest, attempts to identify its homologs in a database of short peptide sequences while also assembling these homologs into complete protein sequences [14] (see the methods section for formal definitions of the gene-centric assembly problem). The simultaneous alignment and assembly algorithm was developed to solve the gene-centric assembly problem and was implemented into a program called GRASP (Guided Reference-based Assembly of Short Peptides) [14]. GRASP outputs both the sequencing reads that are homologous to the reference (similar to other homolog search programs such as BLAST) as well as the corresponding assembled contigs.

GRASP is well suited to solve the gene-centric assembly as well as the annotation problem because it simultaneously alleviates the above mentioned limitations of the direct alignment and *de novo* assembly approaches. First, the sequence similarity is computed between the query and the contig (instead of the individual reads), more accurately reflecting the true homology. Second, alignment is performed between the query and the target (a path in the sequence overlap graph) as the assembly algorithm traverses the sequence overlap graph, estimating the sequence similarity that is later used to guide the traversal towards the correct path. The more informed graph traversal allows for more effective pruning of false paths and meanwhile the identification of low-abundant true homologs. GRASP achieves ~20 % higher recall rate than PSI-BLAST based on simulation, and identifies ~3 times more true homologous reads than PSI-BLAST from a real metagenomics data set without loss of precision. However, GRASP’s computational efficiency is adversely impacted by the assembly module and it requires substantial speedup for applications on large data sets.

In this article we present GRASPx, a computational efficient improvement of GRASP through substantially redesigned algorithm and data structure. Its application as a homology detection program is benchmarked with its predecessor GRASP (to compare running time), BLASTP, PSI-BLAST [1] (NCBI v2.2.28+) and FASTM (v36) [15] (to compare performance). GRASPx is >30X faster than GRASP; it also has a similar running time as PSI-BLAST (with 3 iterations) and therefore is feasible for genome-wide analysis of databases containing tens of millions of sequences. As confirmed by simulation-based benchmark results, GRASPx has a similar accuracy as GRASP, and both of them demonstrate ~20 % higher recall than PSI-BLAST, and ~30 % higher recall than BLASTP and FASTM at the same precision level. GRASPx also demonstrates the best performance among all programs being tested when applied to a real human saliva metagenomic data set. We anticipate that GRASPx will receive wider application for metagenomic analysis because of its high accuracy and substantially improved computational efficiency. GRASPx is freely available at http://graspx.sourceforge.net/.

## Methods

In this section, we first formulate the gene-centric assembly problem and discuss its relationship with the homology detection problem. We then briefly summarize the original GRASP algorithm [14] for solving these two problems. We finally present the intuition and details for the new GRASPx algorithm.

### The gene-centric assembly problem

Here we formally define the gene-centric assembly problem: Given a query protein sequence *q* and a set of short peptide reads *R*, identify a set of contigs *P* (or more precisely *P*^*q*^; we use *P* for simplicity when *q* is clear in the context) such that each sequence *p* ∈ *P* has a sequence similarity with *q* above a certain threshold (e.g. BLASTP E-value cutoff), and that *p* is an assembly of a set of short peptide reads *R*^*p*^ such that *R*^*p*^ ⊆ *R*. Intuitively, such a formulation allows some otherwise low-similarity reads to be assembled with other high-similarity reads and together be identified as homologs of the query *q* [14]. Note that the regular homology search problem that can be solved using the direct alignment approach is a special case of the gene-centric assembly problem with the constraint |*R*^*p*^| = 1. Also note that solving the gene-centric assembly problem immediately solves the homology detection problem: ${\displaystyle \underset{p\in P}{\cup }{R}^p}$ is the set of homolog reads of the query *q* and they can inherit the annotation of *q*. In summary, the metagenomics read annotation problem can be first transformed into homology detection problem and then solved under the gene-centric assembly formulation.

### Summary of the GRASP algorithm

The GRASP algorithm takes the query (or reference) protein sequence as a guide and assembles contigs from the short peptide sequence database such that the alignment score of each contig with the reference sequence meets a pre-specified cutoff. The assembly algorithm of GRASP shares conceptual similarities with sequence overlap-based approaches (e.g. using data structures such as overlap-graph [16], string graph [17], or *de bruijn* graph [18]), except that the graph is not explicitly built in GRASP but that the overlap information is resolved through suffix-array searches whenever needed. The GRASP algorithm that extends towards the C-terminus of the reference sequence (right extension) is presented as follows (extension algorithm towards the N-terminus, i.e. left extension, is analogous to the right extension).

1. *Seeding*: GRASP first identifies exact *k*-mer matches in the reduced-alphabet space [14], which improves sensitivity and selectivity in filtering alignment candidates [3]. It uses the seed in the target (database) sequence as the initial contig to be extended.
2. *Suffix array search*: GRASP searches the fixed-length suffix of the current contig against the suffix array [19] built on the database, and identifies all candidate reads that overlap with the current contig.
3. *Redundancy removal*: The suffix array search returns a list of suffixes that begin with the queried sequence. GRASP traverses the entire list of returned suffixes to identify a set of *maximal extension sequences* (*MESs*), where each MES is defined as a suffix that is not contained in any other suffixes as a substring.
4. *Alignment*: GRASP concatenates the current contig with each of the MESs, and reevaluates the alignment scores between the reference and the extended contigs using a banded Needleman-Wunsch algorithm [20]. It utilizes the recomputed alignment scores as a filter (similar to BLAST’s bit-score drop-off) to select a subset of promising contig extensions. GRASP retains the promising contigs and further extends them by executing the 2^nd^ through the 4^th^ steps iteratively.

### Improvements implemented in GRASPx

#### Prebuilt extension links for maximal extension sequence determination

**Fig. 1:**
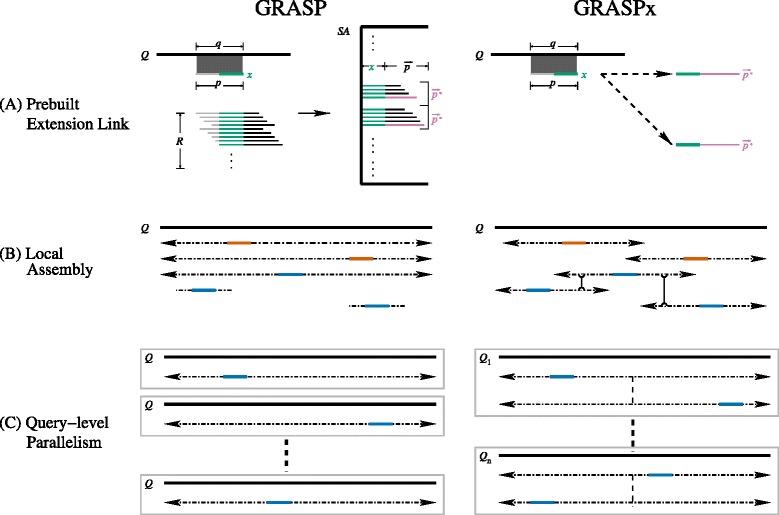
Summary of the major improvements of GRASPx. *Q* is the reference/query sequence. **a** Prebuilt Extension Link: The original GRASP algorithm first searches the suffix *x* (*green*) of the current contig *P* against the suffix array *SA* (which is built on the sequencing read set *R*), and then identifies the MESs $\overrightarrow{p}*$ (*pink*). GRASPx simply follows the pre-built extension links (*black dashed arrows*) to identify all MESs. **b** Local Assembly: The original GRASP algorithm extends the contig until significant score drop-off is observed, which might lead to unnecessary extensions of the random seeds (*orange*). GRASPx extends each seed with a pre-set maximal depth. Homologous contigs (with corresponding *blue seeds*) that are broken by this strategy are re-assembled through a recalibration step based on sequence overlap (highlighted by the vertical double-sided reverse arrows). **c** Query-level Parallelism: GRASP spawns a thread (*gray box*) for each seed extension. GRASPx spawns a thread for each query sequence

The GRASP algorithm identifies MESs through searching the suffix array followed by redundancy removal (Fig. 1a). Note that although the computing time for each step is short (the suffix array search is approximately linear to the read length given a fixed database size, and the redundancy removal is linear to the number of suffixes returned by the search), these steps are performed in each iteration and thus the accumulated computations become a rate-limiting step. To speed up the algorithm, GRASPx pre-builds *extension links* with respect to reads in the given database, allowing for constant-time determination of the MESs for each contig extension (Fig. 1a).

An extension link is defined as a directed edge between two reads, where the sink read is an MES of the source read. It is further required that each source read can be linked to no more than a fix number of sink reads (empirically set to 20 for balanced performance and computational efficiency, data not shown). This is because reads with minor sequence differences (which could due to sequencing errors, single-nucleotide variations, or imperfect repeats) can be recruited through a post-mapping step (see below). In this sense, constructing all extension links is conceptually similar to constructing an alternatively defined string graph (see Additional file 1 for more details). Given the extension links, the algorithm is able to retrieve the MESs directly through following the extension links, therefore bypassing the original rate-limiting suffix array search and redundancy removal steps.

The computational overhead incurred by the extension link is controlled through the development of a novel linear-time construction algorithm (with respect to the short-peptide database size). The algorithm first builds a suffix array and the corresponding Longest Common Prefix (LCP) array from the short-peptide database, followed by a linear traversal of both of the arrays to identify the extension links. In practice, the algorithm runs only slightly slower than the original indexing step (see detailed comparison in the Results section). The extension link construction algorithm and the corresponding pseudocode are presented in Additional file 1. Memory-wise, as the main alignment/assembly module of GRASPx adopts the extension link, it is therefore possible for it to discard the suffix array that is originally required for identifying the MESs. Hence, GRASPx requires similar physical memory as GRASP.

#### Local assembly strategy for each identified seed pair

It has been observed that using a single seed is not selective enough to filter non-homologous seeds for alignment initialization (i.e. a seed match can be identified from a pair of non-homologous sequences by chance); therefore it is desirable to require multiple seeds to improve selectivity. The idea was initially developed in Gapped-BLAST, which requires two seed pairs for alignment initialization [1]. Currently, GRASP only requires a single seed, and its computational efficiency can be further improved based on such an intuition.

However, it is difficult to directly require multiple seeds in GRASPx, because the distance between the seeds in the target sequence is unknown. Estimating such a distance requires the assembly of contigs that contain these seeds, which is itself the central problem to solve here. To circumvent this Catch-22, a *local assembly* strategy is adopted in GRASPx. Specifically, each seed is allowed to be extended with a predefined maximum depth (by default 20 extensions), and the extension is terminated disregarding the drop-off score after reaching the limit. This strategy saves computation time by not extending the non-homologous seeds to the very ends (see Fig. 1b) orange seeds).

Using this strategy, long homologous contigs would be broken into smaller pieces; however, the broken contigs can be re-assembled because multiple seed pairs are expected between homologous sequences (Fig. 1b, blue seeds). On the other hand, the non-homologous contig pieces are unlikely to be re-assembled, as multiple seed pairs rarely exist in non-homologous sequences. A recalibration step is incorporated into GRASPx for re-assembly, which greedily merges the overlapping contigs based on the lengths of the overlaps. The recalibration step also re-evaluates the alignment scores and E-values for the re-assembled contigs.

#### Query-level parallelism for minimizing inter-thread communication

The current implementation of GRASP allows parallel execution; however its efficiency is adversely impacted by the intensive inter-thread communication (a 2-fold speedup was observed while using 4 threads [14]). Specifically, GRASP spawns a thread for the extension for each seed pair, where the thread needs to consult a shared pool of already assembled reads before it can use it for the current extension (if the read is consumed by other threads, the current extension is deemed redundant and subsequently terminated). Correspondingly, the thread also needs to notify the shared pool regarding the reads that have been exploited in the current extension.

In this case, it is expected to minimize the inter-thread communication through spawning a dedicated thread for each query sequence (Fig. 1c). However, it is not a trivial task because GRASP uses a large amount of physical memory to record the *constituent* reads (i.e. the reads that are substrings of the assembled contigs) as well as their placement information. Simply spawning threads at a per-query level would consume a large amount of physical memory. In GRASPx, information regarding the constituent reads is discarded while performing assembly, and subsequently recovered through a post-mapping step that aligns all reads against the assembled contigs (minimum alignment of 60 % of the read length with at maximum 3 substitution errors). This strategy enables GRASPx to spawn multiple threads at a per-query level with reasonable memory consumption. For example, GRASPx requires ~14G of physical memory for searching ~6 million reads with 16 threads, while GRASP requires ~13G for the same search. The overhead incurred by the post-mapping step is trivial compared to that of the assembly stage, as the reads are only mapped to the assembled contigs. For example, GRASPx spends ~36 min for searching 198 marker genes from ~6 million reads, while taking <2 min for post-mapping.

### Description of the GRASPx algorithm

The GRASPx algorithm consists of the following main steps:

1. **Database indexing**: GRASPx pre-builds extension links on a given database, which allows for constant time determination of each MES with respect to a given contig extension. GRASPx performs the indexing step only once.
2. **Seeding**: GRASPx adopts the same strategy as GRASP for seeding, which identifies exact *k*-mer matches between the query and the target database sequences in the reduced-alphabet space.
3. **Extension**: GRASPx simply follows the pre-built extension links to determine all MESs of the given contig. It replaces the second and the third steps of the GRASP algorithm with this computationally efficient look-up step.
4. **Alignment**: GRASPx adopts the same alignment strategy as GRASP (i.e. banded Needleman-Wunsch algorithm [20]). In additional to detecting potential termination criteria, it also terminates the extension if the depth of the extension exceeds a predefined threshold (local assembly). The third and the fourth steps are performed iteratively for each of the identified seed pairs until termination.
5. **Recalibration**: The local alignment strategy would potentially break an otherwise complete contig into smaller pieces. GRASPx attempts to repair the broken contigs using this greedy re-assembly step.
6. **Post-mapping**: To reduce memory consumption, GRASPx does not keep track of the constituent reads for the assembled contigs. To recover this information, GRASPx maps all reads against the assembled contigs to identify the assembled homologous reads. Note that the reads that cannot be assembled due to minor sequencing errors or single-nucleotide polymorphism can be recruited in this stage through allowing mismatches in the alignment.

## Results and discussion

### Data sets

GRASPx was benchmarked with four other homology search programs, i.e. GRASP [14] (not used for large-scale genome-wide benchmark because of its relatively lower computational efficiency), BLASTP, PSI-BLAST [1], and FASTM [15]. For consistency of the benchmark data sets, we selected two data sets that were previously used to benchmark GRASP [14]:

- **DS1**: This simulated data set was generated from 20 marine microbial genomes (with staggered abundances; details available from reference [12]) using WGSIM [21] at 10X coverage with an expected length of 100 bp and an error rate of 1 % (for the Illumina technology). Short peptides were identified from the simulated reads using FragGeneScan [11], resulted in 6,273,043 short peptide reads.
- **DS2**: This real data set was generated from a human saliva sample by the Human Microbiome Project [22, 23]. It was downloaded from NCBI’s Sequence Read Archive (SRA) with accession number SRS013942. Short peptides were also called from the nucleotide reads using FragGeneScan [11]. The resulting database contained 12,036,685 short peptide reads.

### Parameters

All experiments were performed on an in-house server equipped with four Intel Xeon X7350 @2.93GHz processors and 256GB physical memory. GRASPx, GRASP, BLASTP, PSI-BLAST, and FASTM were invoked using their default sets of parameters, or are otherwise detailed as follows. PSI-BLAST was run with three iterations. FASTM was run using the BLOSUM62 scoring matrix with gap opening and gap extension penalties of -11 and -1, respectively (by specifying the ‘-s BL62’ argument), to match the settings used by the other programs.

Below we detailed the set of default parameters that are configured for GRASPx, which were used in all experiments presented in this article. GRASPx requires a 6-mer perfect match between the query and the target in the GBMR10 reduced alphabet [3] for identifying seed pairs. It further requires that the alignment score between the two sequences of the seed pair is at least 6 * 0.7 * *a*, where *a* is the mean of the diagonal scores of the scoring matrix in use. GRASPx adopts a minimum overlap of 10aa between reads during the extension link construction phase; and it extends the assembly with a maximum depth of 20 towards each direction (for local assembly). For the alignment module, GRASPx utilizes the BLOSUM62 matrix with gap opening and extension penalties of -11 and -1, respectively. GRASPx adopts the same approach as in BLAST to compute the bit score and E-value, and it also uses the same drop-off score cutoff as BLAST, i.e. 25 bits. The default band size for sequence alignment is 40.

### Running time improvement of GRASPx

**Fig. 2:**
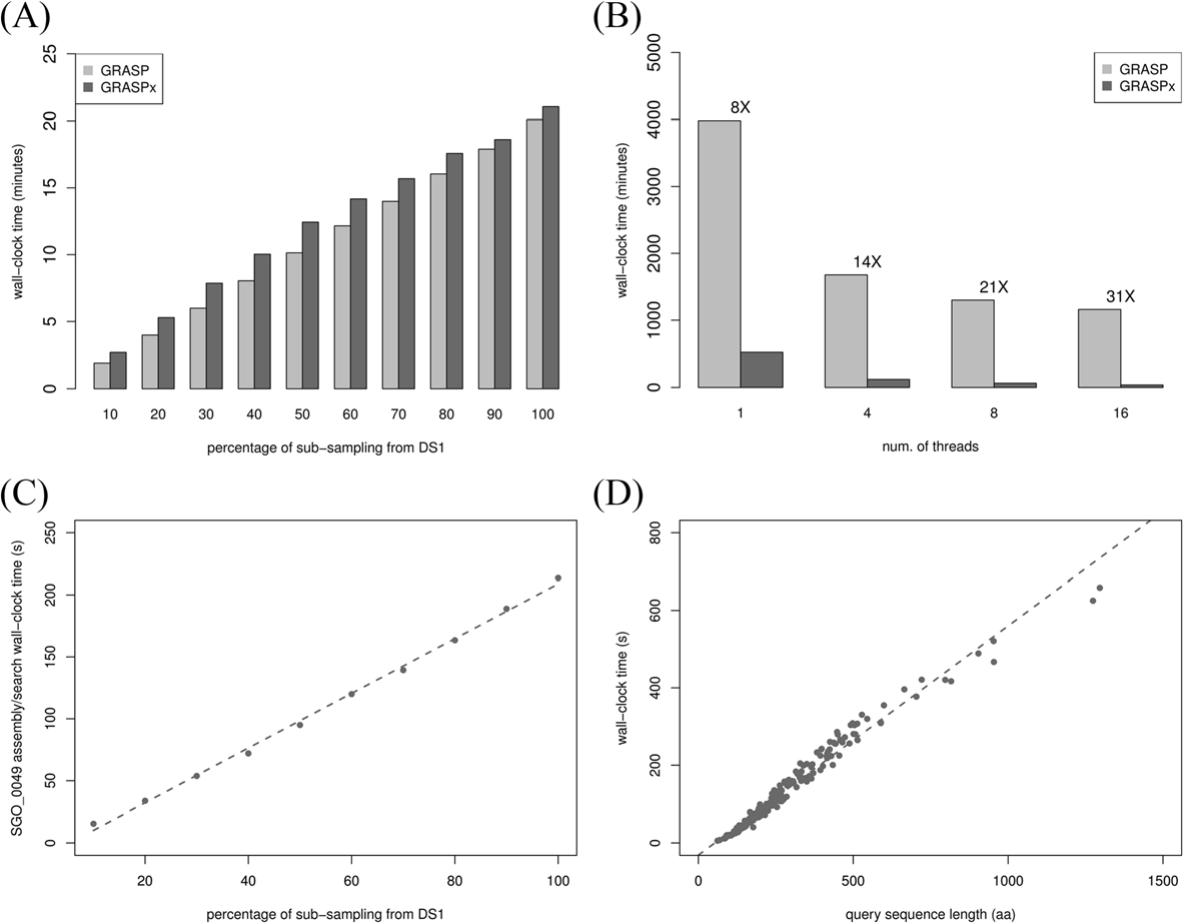
Running time of GRASPx. Running time evaluated by searching the 198 *D. sp. CBDB1* Amophora2 marker genes against DS1. **a** Total indexing time of GRASP and GRASPx on simulated data sets that were randomly sampled from DS1 with various sizes. **b** Total runtime of GRASPx and GRASP (numbers above bars indicate speedups) when run with the corresponding number of threads. **c** GRASPx’s runtime (single-threaded) for searching a single protein sequence SGO_0049 (344 aa) against simulated data sets that were randomly sampled from DS1 with various sizes. **d** GRASPx’s runtime (single-threaded) for searching Amphora2 marker genes against DS1

Both the indexing time and the alignment/assembly time of GRASPx were measured and compared with those of GRASP. The wall-clock indexing time of both programs on databases with different sizes (generated by random sub-sampling with different proportions from DS1) are shown in Fig. 2a. The indexing time of GRASPx is longer than that of GRASP, potentially due to the additional works that are required to create the extension links. However, the actual indexing time for both programs remains comparable. The wall-clock alignment/assembly time of GRASPx for searching 198 *Dehalococcoides sp. CBDB1* marker genes [24] against DS1 is shown in Fig. 2b. The results show that GRASPx is significantly faster than GRASP in the alignment/assembly phase. The speedup is more significant when more threads are used (8X speedup when both programs were run with a single thread and 31X speedup when both run with 16 threads), showing the advantage of the redesigned parallelization strategy of GRASPx. The results indicate that GRASPx algorithm achieves significant alignment/assembly speedup at a cost of marginally increased indexing time.

The empirical time complexity of GRASPx (for the alignment/assembly phase) is shown by searching a single protein sequence against different databases with various sizes (Fig. 2c), as well as searching different protein sequences with various sizes against a fix-sized database (Fig. 2d). It is observed that the running time of GRASPx grows linearly in both cases, indicating that GRASPx is scalable for large-scale analysis.

Finally, the running time of GRASPx on searching a much larger number of query protein sequences (~2000) against DS2 was summarized in Table 1. By comparing the GRASPx and the PSI-BLAST running time (running time of BLASTP and FASTM are not included because of their lower search performances), it is observed that PSI-BLAST requires less (approximately 1/5) CPU time compared to GRASPx for the searches. However, GRASPx is capable of more effectively utilizing multiple threads than the current implementation of PSI-BLAST, resulting in a comparable wall-clock running time.

**Table 1:** Running time comparison of GRASPx and PSI-BLAST on DS2

Query	Num.	GRASPx			PSI-BLAST		
genome	prot.	CPU	Wall	Perc. CPU	CPU	Wall	Perc. CPU
SGO	2051	148h55m	9h36m	1553 %	29h20m	9h52m	304 %
PAC	2297	157h40m	10h01m	1574 %	26h27m	8h35m	315 %

‘Query genome’ indicates the genome from which the query sequences were obtained; SGO: *Streptococcus gordonii*; PAC: *Propionibacterium acnes*. ‘Num. prot.’ indicates the number of protein sequences that are annotated in the corresponding genome and used as the queries. ‘CPU’ and ‘Wall’ indicate the CPU time and wall-clock time for the corresponding program to search all query proteins sequences against DS2, respectively. ‘Perc. CPU’ indicates the percentage of CPU used for the search (both programs were assigned with 16 CPUs; PSI-BLAST only used 4 CPUs at maximum)

### Performance of GRASPx on DS1

GRASPx was benchmarked with GRASP (post-mapping step included for optimized performance, as described in [14]), BLASTP, PSI-BLAST, and FASTM on DS1.

Define the ground-truth set of homolog reads with respect to a query sequence *q* as follows. Recall that DS1 was simulated through random sampling of reads from the 20 marine microbial genomes described in [12]; define *G* as the concatenation of the 20 reference genomes. Also note that in cases of simulation, it is trivial to record where (in terms of a genomic interval in *G*) a read was sampled. Let *I* (more precisely *I*^*G*^; we used *I* for simplicity when *G* is clear in the context) be an arbitrary set of genomic intervals in *G*, and correspondingly denote the set of reads that were sampled from *I* as *R*^*I*^. (In practice, *R*^*I*^ includes reads that have >60 % of their sequences sampled from *I*.) For a given query sequence *q*, define its homolog intervals in *G* through searching *q* against *G* using TBLASTN with E-value cutoff 10^-3^; denote the set of homolog intervals as *I*^*q*^. The ground-truth homolog read set for *q* is thus defined as ${R}^{I^q}$.

Subsequently, define TP (true positive) as the reads that are both in ${R}^{I^q}$ and identified by the search (using a specific program), FP (false positive) as the reads that are not in ${R}^{I^q}$ but identified by the search, and FN (false negative) as the reads that are in ${R}^{I^q}$ but not identified by the search. Recall, precision, and F-measure can subsequently be computed as:

$$ recall=\frac{TP}{TP+FN}, precision=\frac{TP}{TP+FP},\mathrm{and}\ F=\frac{2* recall* precision}{recall+ precison}, $$

The performances of all programs were investigated with different E-value cutoff ranging from 10^-10^ to 10, which were then plotted as the Receiver Operating Characteristics (ROC) curves. Subsequently define the Area Under the ROC Curve (AUC) as:

$$ AUC={\displaystyle \sum_k\frac{\left( recal{l}_{k+1}+ recal{l}_k\right)\left( precisio{n}_k- precisio{n}_{k+1}\right)}{2}}; $$

where *recall*_*k*_ and *precision*_*k*_ are the recall rate and precision rate for the *k*th ascending E-value cutoff, respectively.

**Fig. 3:**
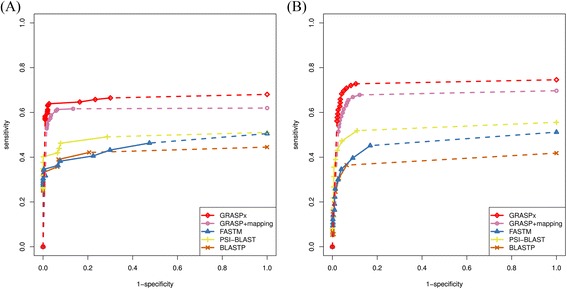
Performance of GRASPx on the simulated data set DS1. Performances were measured for GRASPx, GRASP + mapping, FASTM, PSI-BLAST (with 3 iterations), and BLASTP on searching (**a**) 16 glycolysis related *D. sp. CBDB1* genes and (**b**) 198 *D. sp. CBDB1* Amphora2 marker genes against DS1. *Dashed lines* indicate extrapolated performances. GRASPx shows marginally improved performance over GRASP + mapping, and both of them significantly outperform the other programs

Two small sets of query protein sequences were first used to measure the performances of the programs. The first query set contained 16 *D. sp. CBDB1* genes participating in the glycolysis pathway (KEGG [25] pathway ID: KO00010). The second query set contained 198 *D. sp. CBDB1* marker genes that were collected in the Amphora2 database [24]. The ROC curves for glycolysis and Amphora2 protein searches are shown in Fig. 3a and b, respectively. The results confirm that GRASPx has a comparable performance with the original GRASP (with mapping, denoted in Fig. 3 as “GRASP + mapping”). It is also observed that GRASPx has improved the recall rate of PSI-BALST by at least 20 % at the same precision level for both experiments, suggesting potential applications of GRASPx in both functional (e.g. glycolysis pathway) and taxonomic (e.g. Amphora2 marker genes) analysis of metagenomics sequencing data.

The performance of GRASPx was further benchmarked on searching all 1458 protein sequences annotated in the *D. sp. CBDB1* genome (GRASP was not included for its lower computational efficiency) against DS1. Homologous reads with respect to each query protein sequence were further classified as *close* (TBLASTN E-value cutoff 10^−10^), *moderate* (TBLASTN E-value cutoff 10^−3^) and *remote* (TBLASTN E-value cutoff 10) homologs reads. The performances (the optimal F-measure and the AUC) of the search programs are summarized in Table 2 (with performances of several important metabolic pathways highlighted). For most of the GRASPx searches, the optimal performances were achieved with E-value cutoff ranging from 10^−1^ to 10^−3^, which is consistent with the results shown in Fig. 3. GRASPx achieves the highest performance on the majority of the searches of individual pathways as well as the overall search. GRASPx shows more significant improvement when searching remote homology. For example, the optimal F-measure and AUC of GRASPx is 8 % higher (i.e. on average 8 % higher recall and precision rate) than PSI-BLAST when detecting remote homology, compared to 5 % higher when detecting close homology.

**Table 2:** Performances of four programs on searching all 1458 *D. sp. CBDB1* protein sequences against DS1

Pathway	BLASTP			PSI-BLAST			FASTM			GRASPx		
	C^4^	M^5^	R^6^	C	M	R	C	M	R	C	M	R
TCA cycle^1^	0.61	0.38	0.37	0.69	0.44	0.42	0.57	0.32	0.28	0.88	0.64	0.59
^2^	0.66	0.44	0.42	0.75	0.55	0.52	0.44	0.10	0.08	**0.88**	**0.74**	**0.69**
Pentose	0.67	0.71	0.64	0.76	0.76	**0.68**	0.54	0.52	0.40	**0.85**	**0.78**	0.64
phosphate	0.61	0.62	0.50	0.71	0.72	0.61	0.41	0.41	0.26	**0.87**	**0.84**	**0.74**
Fructose	0.61	0.48	0.38	0.53	0.52	0.42	0.38	0.38	0.26	**0.75**	**0.66**	**0.53**
mannose	0.50	0.51	0.38	0.56	0.58	0.45	0.38	0.38	0.22	**0.80**	**0.75**	**0.64**
Pyruvate	0.55	0.41	0.40	0.64	0.48	0.45	0.42	0.25	0.22	**0.72**	**0.56**	**0.52**
	0.59	0.43	0.40	0.69	0.56	0.53	0.32	0.08	0.07	**0.78**	**0.66**	**0.63**
Methane	0.57	0.38	0.27	0.63	0.43	0.31	0.56	0.31	0.21	**0.75**	**0.56**	**0.44**
	0.66	0.49	0.35	0.70	0.56	0.42	0.57	0.28	0.12	**0.82**	**0.67**	**0.53**
Nitrogen	0.54	0.53	0.51	0.56	0.55	0.54	0.05	0.05	0.05	**0.59**	**0.59**	**0.58**
	0.35	0.34	0.34	0.53	0.52	0.51	0.01	0.01	0.01	**0.69**	**0.69**	**0.68**
Sulfur	0.79	0.72	0.68	0.83	**0.75**	**0.70**	0.56	0.48	0.38	**0.84**	**0.75**	0.68
	0.75	0.65	0.57	0.80	0.71	0.64	0.22	0.15	0.12	**0.86**	**0.78**	**0.73**
Overall^3^	0.59	0.51	0.30	0.66	0.57	0.36	0.52	0.43	0.22	**0.71**	**0.64**	**0.44**
	0.65	0.59	0.40	0.72	0.65	0.47	0.59	0.49	0.23	**0.77**	**0.72**	**0.55**

^**1**^: The first row indicates the area under curves (AUC) for the corresponding programs. ^**2**^: The second row indicates the F-scores for the corresponding programs. ^**3**^: The overall performance is calculated on all 1458 protein sequences annotated in the *D. sp. CBDB1* genome. ^**4**^: ‘C’ indicates close homologs (defined by TBLASTN E-value cutoff 10^-10^). ^**5**^: ‘M’ indicates moderate homologs (defined by TBLASTN E-value cutoff 10^-3^). ^**6**^: ‘R’ indicates remote homologs (defined by TBLASTN E-value cutoff 10). The highest performances among all programs are bolded

### Performance of GRASPx on DS2

For the real data set DS2 where the ground-truth homolog intervals cannot be defined (no complete reference genome available for real data sets), we alternatively measured TP (instead of recall rate) and precision rate of the programs. Given the query *q* and its corresponding Pfam model *F*^*q*^ retrieved from KEGG [25], define TP for a given search as the identified reads that can be classified as a member of *F*^*q*^ (using HMMER3 [26] with the default trusted E-value cutoff 10^-2^), or the identified reads that are substrings of some assembled sequences *p* such that *p* can be classified as a member of *F*^*q*^ (in practice we allow up to 3 substitution errors and require that >60 % of the read aligned to *p*). Also define FP as the identified reads that are not TP. Precision rate can be subsequently computed in the same way as for DS1.

**Fig. 4:**
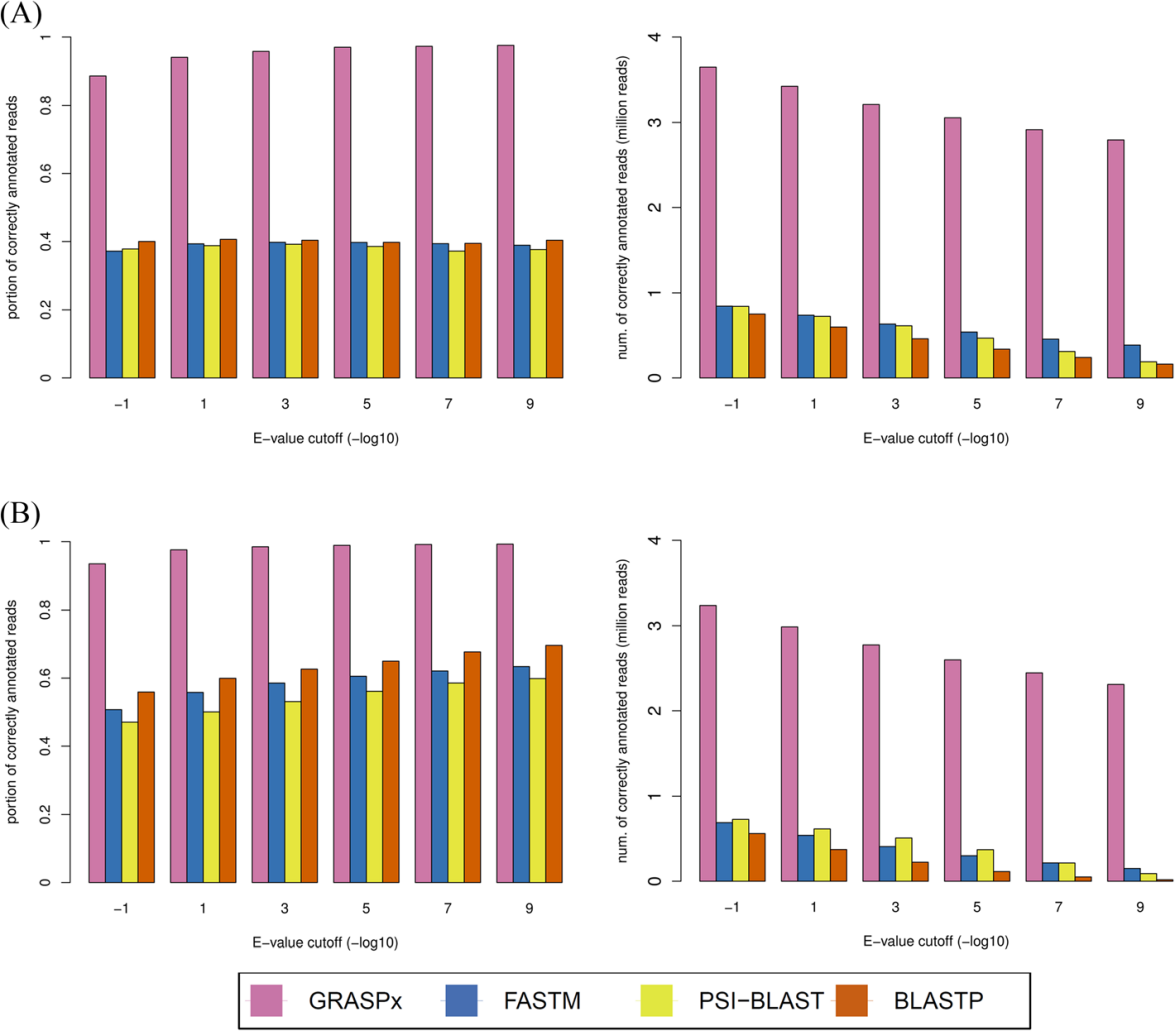
Performance of GRASPx on the real data set DS2. Performances were measured for GRASPx, FASTM, PSI-BLAST, and BLASTP on searching (**a**) all (2051) protein sequences annotated in the *S. gordonii* genome and (**b**) all (2297) protein sequences annotated in the *P. acnes* genome against DS2. *Left panel*: precision rate. *Right panel*: number of true positive (TP) predictions

Annotated protein sequences from *Streptococcus gordonii* and *Propionibacterium acnes* genomes (2051 and 2297 sequences respectively) were used as queries for this experiment. The search results for the two sets of query proteins are shown in Fig. 4a and b, respectively. The left panel of Fig. 4 shows the precisions of the corresponding experiments while the right panel shows the raw numbers of TPs. As shown in the left panel of Fig. 4a and b, 88.5 to 97.5 % (corresponding the E-value cutoff ranging from 10 to 10^−9^) of the reads identified by GRASPx corresponds to true homologous reads, compared to only ~40 % of the other programs. The high precision of GRASPx indicates that it is capable of assembling more reads into annotatable contigs (which can be unambiguously classified as a member of the corresponding protein family). On the other hand, many of the individual homologous reads predicted by the other programs are difficult to annotate, potentially because they are not sampled from the conserved domains of the protein family. The right panel of Fig. 4 shows that GRASPx is also capable of identifying much more reads than the other programs (at least 4 times). In summary, the results suggest that GRASPx can identify more true homologous reads from real metagenomic data sets with higher precision rate than other programs; and at the same time assemble them into annotatable contigs that significantly facilitate downstream functional analyses.

### Utility of GRASPx in pathway reconstruction

Here we demonstrate the utility of GRASPx in pathway reconstruction. MinPath [27] was used to reconstruct pathways using, respectively, GRASPx and PSI-BLAST identified homologous reads while searching all *P. acnes* encoded proteins against the human saliva data set DS2. The *P. acnes* genome was chosen because it is known to be rare from healthy human saliva samples; and it simulates a challenging scenario where one happens to choose the protein sequences of low-abundant bacteria as references. GRASPx identified 2,326,282 reads with E-value cutoff 10^−10^; MinPath reconstructed 73 pathways using these reads. On the other hand, PSI-BLAST identified 24,739 reads for the same search with E-value cutoff of 10^−10^; MinPath reconstructed 71 pathways from these reads. The set of 73 pathways reconstructed using GRASPx predicted reads fully contains the set of 71 pathways reconstructed using PSI-BLAST predicted reads.

The two pathways that were uniquely identified using GRASPx predictions are KO00071: fatty acid degradation and KO00520: amino acid sugar and nucleotide sugar metabolism. They have been reconstructed from human saliva metagenomics data independently by multiple research groups [28–30]. The reconstruction of both pathways suggests high detection power of GRASPx: it is possible to use low-abundant and distantly related sequences as the references, and accurately identify homologous sequences and estimate their abundances. Such an advantage allows for functional analysis of metagenomic sequences without requiring complete reference genome sequences.

While the results can be used to establish high recall rate for GRASPx, it is possible that PSI-BLAST did not identify reads correspond to these pathways simply because there is no sequence that is closely-related (by close we mean with PSI-BLAST E-value cutoff 10^−10^, which is used for the search) with *P. acnes* exists in DS2. To investigate such a possibility, it was found that the two pathways are not reconstructed because PSI-BLAST did not identify any homologous reads for two querying *P. acnes* proteins, namely pac:PPA1632 (long-chain acyl-CoA synthetase, involved in pathway KO00071) and pac:PPA0343 (glucose-1-phosphate thymidylyltransferase, involved in pathway KO00520). To verify whether DS2 contains closely-related homolog sequences of *P. acnes*, the GRASPx identified contigs were aligned to the corresponding queries using BLASTP. The best E-value achieved for pac:PPA1632 was 2 × 10^− 94^; and for pac:PPA0343 was 2 × 10^− 172^ (see Additional file 1). While it is still unclear that, in biological sense, whether these contigs are closely related with *P. acnes*; these contigs indeed share high sequence similarity with the queries and thus should be detected for the given E-value cutoff. The results confirm that the reconstruction of pathways of KO00071 and KO00520 is due to the high recall rate of GRASPx, rather than the lack of closely-related homologs in the database.

MinPath only predicts the presence/absence of the pathways and ignores their actual abundances [27], such that it is recalcitrant to less-sensitive homology predictions and has successfully reconstructed majority of the pathways even using PSI-BLAST predictions. On the other hand, while using abundance-aware pathway reconstruction tools (e.g. HUMAnN [31]), the reconstruction results for using GRASPx and PSI-BLAST predictions could be even more significant. Meanwhile, the performance of these abundance-aware pathway reconstruction tools could also be improved by using GRASPx for that it more accurately estimates the true abundances of the proteins of interest [31].

## Conclusions

In this work we present a computational efficiency improvement of the simultaneous alignment and assembly algorithm. The improvement is made possible by three technical redesigns of the original algorithm. The construction of the extension links pre-computes the overlap information, speeding up the path extension step of the assembly module. The use of the local assembly strategy adopts a similar filtering heuristic implemented in the Gapped-BLAST, alleviating the bottleneck of searching extremely long query protein sequences. Finally, the re-implemented parallelization strategy allows for more effective usage of multi-core resources, rendering the search of multiple queries at a time possible. The resulting program is named GRASPx.

In conclusion, GRASPx was developed as a simultaneous alignment and assembly program suitable for metagenomic data analysis in this work. GRASPx is capable of performing reference-based search (similar to the BLAST suite, the FASTA suite, and RAPSearch etc.) as well as gene-centric assembly of the identified reads. According to our benchmark test, GRASPx is more than 30X faster than its predecessor GRASP while keeping the same level of performance. GRASPx has a similar running time as PSI-BLAST, enabling genome-wide homolog detection on large metagenomic data sets with superior sensitivity and specificity. Practically, GRASPx allows assembly and search of homologous reads with respect to all protein sequences encoded in a bacterial genome against a moderate-sized metagenomic data set (e.g. ~40 million reads and ~100 bp per read) within approximately 12 h using 16 threads. We expect that GRASPx would substantially improve metagenomic applications such as gene abundance estimation and pathway reconstruction.

## Additional files

Additional file 1: Algorithm for constructing extension links and BLASTP alignments between selected *P. acnes* proteins and their predicted homologous contigs. The file contains detailed algorithm and pseudo-code for linear construction of extension links. The file also contains NCBI BLASTP alignments of two *P. acnes* proteins, whose homologous reads were not identified by PSI-BLAST from DS2 but were identified by GRASPx. (PDF 813 kb)
