# Supplementary material for: GRASPx: efficient homolog-search of short peptide metagenome database through simultaneous alignment and assembly
Source: BMC Bioinformatics. 2016 Aug 31;17(Suppl 8):283. doi: 10.1186/s12859-016-1119-1 (PMC5009819; doi:10.1186/s12859-016-1119-1)
Supplement: Additional file 1: — Algorithm for constructing extension links and BLASTP alignments between selected P. acnes proteins and their predicted homologous contigs. The file contains detailed algorithm and pseudo-code for linear construction of extension links. The file also contains NCBI BLASTP alignments of two P. acnes proteins, whose homologous reads were not identified by PSI-BLAST from DS2 but were identified by GRASPx. (PDF 813 kb) [file 12859_2016_1119_MOESM1_ESM.pdf]

Additional File for “GRASPx: Efficient Homolog-Search of Short Peptide Metagenome Database through Simultaneous Alignment and Assembly”

Cuncong Zhong, Youngik Yang, Shibu Yooseph*

Informatics Department, J. Craig Venter Institute, La Jolla CA 92037

*Corresponding author

Construction of extension links in linear time with suffix and LCP array

In this section, we focus on presenting the algorithm for constructing the extension links for the forward extension direction (i.e. towards the C-terminus); algorithm for the opposite direction can be derived analogously. The input is the metagenomics sequencing read database R, where the ith read in R is denoted as $R[i]$. Suffix array for R can be constructed using C++ library *libdivsufsort* (https://code.google.com/p/libdivsufsort/). Denote the resulting suffix array as SA. SA is an array of lexicographically sorted suffixes of reads in R. Denote the ith suffix in the array as $SA[i]$ and its length as $|SA[i]|$. During the construction of the suffix array, the longest common prefix (LCP) information is also computed for all adjacent suffixes (i.e. LCP between $SA[i-1]$ and $SA[i]$ for any $2 \le i \le N$, where N is the size of SA). For simplicity denote such LCP information as $LCP[i-1,i]$. The purpose is to construct the extension links using the suffix array SA and the LCP array LCP through a single traversal of SA and LCP.

The major intuition of the algorithm is summarized as the follows. Denote the shortest super string of two reads $R[i]$ and $R[j]$ as $\hat{u}(i,j)$, given that the prefix of $R[j]$ perfectly matches the suffix of $R[i]$ with a length of at least l. Define extension link formally as the follows: an extension link from $R[i]$ to $R[j]$ exists if there is no read $R[k]$ such that $\hat{u}(i,k)$ is a proper super string of $\hat{u}(i,j)$. By the lexicographical ordering of SA, it is clear that if such $R[k]$ exists, $R[k]$ must be ranked after $R[j]$ in SA. Also, the corresponding suffix (by removing the sequence of $R[i]$ from $\hat{u}(i,j)$) of $R[j]$ must be a proper prefix of the corresponding suffix (by removing the sequence of $R[i]$ from $\hat{u}(i,k)$) of $R[k]$, because $\hat{u}(i,k)$ is a proper super string of $\hat{u}(i,j)$. Combining both observations, it is clear that the sink read of the extension link is the lowest-ranked (i.e. with larger SA index) suffix that satisfies such a property. The algorithm is presented as follows.

The algorithm begins by identifying an initial set of source reads through scanning for suffixes that are exactly l-long (10aa by default); once encountered (could be one or more such consecutive suffixes that have the same l-long sequence), the algorithm takes all corresponding reads as the initial source reads and proceeds to search the sink reads. Let the suffix that is currently being traversed be $SA[i]$. Three cases are possible. First, we have $LCP[i-1,i] = |SA[i-1]|$ and $LCP[i-1,i] \geq l$. It indicates that $SA[i-1]$ is not an MES as it is contained by $SA[i]$; however it is unclear whether $SA[i]$ is an MES (e.g. $SA[i]$ might be contained by $SA[i+1]$). In this case nothing needs to be done. Second, we have $LCP[i-1,i] < |SA[i-1]|$ and $LCP[i-1,i] \geq l$. This case indicates that $SA[i-1]$ is not contained in $SA[i]$, and therefore $SA[i-1]$ is an MES (note that suffixes are lexicographically sorted in SA). Connect all source reads with the read that correspond to $SA[i-1]$ with extension links. Finally, we have $LCP[i-1,i] < l$. It indicates that there is no other suffix in SA that contains the l-long suffix of the source reads (again because of the lexicographically sorted property). The algorithm clears existing source reads and continues to search for the next set of source reads. The algorithm traverses the suffix array exactly once and therefore guarantees a linear time complexity. The pseudo-code of the algorithm is summarized in Figure S1. Note that in the pseudo-code, $SA[i].rid$ is used to represent the corresponding read identifier for the suffix $SA[i]$.

Here we discuss the difference between the extension link and the string-graph concept. First of all, the extension link represents *maximal* extension (by definition) of the current contig while the string graph represents *minimal* extension (by removing transitive edges) of the current contig. Therefore, the use of extension link would lead to faster (maximal extension length indicates minimum number of alignment extensions, where is triggered with certain computation overhead) but less specific search algorithm. We also note that the extension link construction algorithm only requires l-mer overlap between two reads $R[i]$ to $R[j]$, but does not guarantee perfect overlap between the prefix of $R[j]$ and the suffix of $R[i]$. Instead, it only assumes that if l is set to be large enough, then these sequences overlap perfectly with a high probability. The resulting effect is also a faster but less specific extension link construction algorithm. The intuition under such design is to ensure faster search/indexing time, as computational efficiency is the major concern of the current implementation of the algorithm. The low specificity issue is amended using sequence similarity information during the alignment/assembly phase; i.e. it is unlikely that random sequences can be concatenated into a significant homolog of the query, in which case the extensions based on false extension links will receive low alignment score and be dropped in an early stage of the alignment/assembly algorithm.

```
ConstructExtensionLink( l , SA , LCP ):
----------------------------------------------------------------
K = Ø             // K contains all source reads
for i in 2...N do:
        if |SA[i]| == l and ( K == Ø || LCP[i-1,i] == l ) do:
                // same suffix, recruit as potential source reads
                K = K ∪ SA[i].rid
        else if LCP[i-1,i] == |SA[i-1]| and LCP[i-1,i] ≥ l do:
                // do nothing
        else if LCP[i-1,i] < |SA[i-1]| and LCP[i-1,i] ≥ l do:
                // SA[i-1].rid is a maximal extension sequence
                // connect all source reads with it
                for j in K do:
                        Create extension link from j to SA[i-1].rid
                endfor
        else if LCP[i-1,i] < l do:
                // no further extension link is possible for the current
                // overlapping sequence, clear set K
                K = Ø
        endif
endfor
// the last suffix could also be maximal extension sequence
if K != Ø do:
        for j in K do:
                Create extension link from j to SA[N-1].rid
        endfor
endif
```

Figure S1: Pseudo-code of the extension link construction

BLASTP alignment for selected *P. acnes* proteins and their corresponding homologous contigs predicted by GRASPx

Sequence ID: lcl|62553 **Length:** 617 **Number of Matches:** 1

Range 1: 19 to 612 Graphics   Next Match   Previous Match

Score	Expect	Method	Identities	Positives	Gaps
295 bits(754)	2e-94	Compositional matrix adjust.	186/607(31%)	301/607(49%)	18/607(2%)

```
Query  23    NFAAMLDRQADAHPNRIAFLTPSGPDDEPNTWLPMTFAEFRRQAHEVAAGLMEFGLPREG  82
             +F   + +QA    N  A         E   W  +++  F+ Q ++++  L+   +  +
Sbjct  19    HFVHRIQQQAKTRANMTALRYK-----EHGLWRDISWKNFQEQLNQLSRALLAHSIGVQD  73

Query  83    RVALLSGTRTEWIIADMAISCAGGATTTIYPNSGPEEASFILVDSHSSILFVDSTAQVAK  142
             ++A+ +     W IAD+A       T  IY  +  ++A FIL  +   ILFV    Q
Sbjct  74    KIAIFAHNMERWTIADIATLQIRAITVPIYATNTAQQAEFILNHADVKILFVGDQEQY--  131

Query  143   IQGRPEVDAVVRHIISFVDDSEQTGVSDDRLTTM-ADVIAHGRRLAAEPELVRRMIDSI  201
              +   E+      +   V   EQ  +S++ L+    D+I  G      E E   R+ +
Sbjct  132   -EQALEIAHQCPQLQKIVAMKEQIQLSENTLSCHWEDLIQLGTEEFKTEFE--TRLANKT  188

Query  202   EPDDLCTLIYTSGTTGTPKGVELTHQAWTYMGQAWK-SLDMFRGGDIHLLWLPLSHAFGK  260
               DDL T+IYTSGTTG PKGV L +    +  +A   +LD+    ++ L +LP SH F +
Sbjct  189   -MDDLFTIIYTSGTTGEPKGVMLDYNNLAHQLEAHDIALDV-NQDEVSLSFLPFSHIFER  246

Query  261   CLIAICVEIGITQAIEPRIPRLARSLGEVKPRVMCGVPRIFEKIRAGVMTAYPQG-RLAS  319
               +A  +  G          ++  +L E++P +MC VPR +EKI A V+    +  +L
Sbjct  247   AWVAYVLHRGAILCYLEDTNQVRSALTEIRPTLMCAVPRFYEKIYAAVLDKVQKAPKLRQ  306

Query  320   RVSRWAFATGRDVQQYRRAGDRLPVTVAAKLKIADALVFSTLRRKLGG-IEFMICGGAKL  378
              +  WA + G+     R     +P  +  +  +AD LV S LR+ LGG I+ M CGGAKL
Sbjct  307   IMFHWAISVGQKHFDLRANNKAIPFLLKKQFALADKLVLSKLRQLLGGRIKMMPCGGAKL  366

Query  379   SEQVQQWFFSAGIPIVEGYGATEIGAVAFFSGPTAIRSGTVGPVAPGCLARIAEDGEVLV  438
                +  +F S GI I  GYG TE  A             ++G + P    +I E+ E+LV
Sbjct  367   EPTIGLFFHSIGINIKLGYGMTETTATVSCWDDHHFNPNSIGKLMPNAEVKIGENNEILV  426

Query  439   SGPIVARGYHNLPEKTAKAFT-DGWFHTGDIGEFDEKNYLRITDRKRDLFKTSGGKYVAP  497
              G +V +GY+  PE+TA+AFT DG+  TGD GEFDE+  L ITDR ++L KTS GKY+AP
Sbjct  427   RGGMVMKGYYKKPEETAQAFTEDGFLKTGDAGEFDEQGNLFITDRIKELMKTSNGKYIAP  486

Query  498   QKVEATLMANCPYLSNAVVLGEGHKYAVALLTLDRDALMTWGKRHGKADASYAELTADPR  557
             Q +E  +  +  ++    ++ +  KY  AL+    D++  + K+    D    EL
Sbjct  487   QYIEGKIGKD-KFIEQIAIIADAKKYVSALIVPCFDSVEEYAKKLNIKDQDRMELLKHSE  545

Query  558   VRRSIQWYVDRANSRLERWETVKKFAILDHDLTEDSHSVTTSLKVRRGVVAEKYAYLLDE  617
             + +  +  ++     L  +E VKKF +L    +     +T +LK+RR V+ E+Y +++D
Sbjct  546   IIKMFEQRIESLQKELAHFEQVKKFTLLSQAFSVKLGEITPTLKLRRKVIMERYRHIIDS  605

Query  618   MFADEND  624
             M+++  +
Sbjct  606   MYSNNKE  612
```

Figure S2: NCBI BLASTP results for aligning the query sequence pac:PPA1632 (KEGG orthology: K01897) from the *P. acnes* genome against the best contig assembled by GRASPx.

Sequence ID: lcl|16043 Length: 629 Number of Matches: 1

Range 1: 1 to 628 Graphics ▼ Next Match ▲ Previous Match

Score	Expect	Method	Identities	Positives	Gaps
500 bits(1288)	2e-172	Compositional matrix adjust.	279/635(44%)	397/635(62%)	16/635(2%)

```
Query  27   LSWLAFNERVLDLARDTERIPLLERAKFLAIFSSNLDEFFMVRVAGLKRRIDAGVAVPSV  86
            LSWL FN+RV+  A DT   PL+ER +F+AI SSNLDEFFM+RVAGL+ +   G+
Sbjct  1    LSWLKFNQRVVLEAVDTSN-PLMERLRFIAIASSNLDEFFMIRVAGLRHQAMNGIVKYDA  59

Query  87   AGMLPRELHDAILARTHDLVSEQSRVFAEEVRPGLVDEGIEILRWAELSDDEKGRMRTLF  146
            A M  +    AI      LVS QS  + + V   L   G       +L    K  +R  F
Sbjct  60   AHMDAKAQLKAIDESVQRLVSMQS-TYLKNVLCELESHGFYFTYPEQLDVKSKAWLRHYF  118

Query  147  SERIFPILTPLAVDPSHPFPYIRGLSINLAV---MLANPITGAEQFARVKVPSVLPRLVN  203
             E I+P++TPLAVD  HPFP++   +IN  V    +    T   + A + +PSVL R++
Sbjct  119  EEHIYPVVTPLAVDSGHPFPFLTNHTINAIVRIFQIQEDGTKDYKIAILPIPSVLDRIIE  178

Query  204  L-----GEGRFLPLEEIISRHLDQLFTGMHVLQHTTFRVTRNEDLEVEEDDAENLLFALE  258
            +      E RF+ LE++I+ + +Q F G  +  +  FR+TR+ DLE++E++A +LL  +E
Sbjct  179  IPSRGNKEHRFVYLEDVITYYANQFFQGYGIEDYMVFRITRDADLEIDEEEATDLLSEVE  238

Query  259  KELLRRKVGRPPVRLEVQDDISAEMLELLTRELDIRDKEVFRLPAPLDLTGLFSLADV-D  317
              L RR+ G   VRLEV  ++  ++L+ +   +++  K+V+R+   LD    F+  +
Sbjct  239  ASLRRRRRG-DAVRLEVCGEVKDDLLDFVLTSVELEPKDVYRIDGHLDCRMYFNFCNYPG  297

Query  318  RDDLSYPNFLPITHPHLAEVETARPADMFAAIRRRDVLVHHPYDSFATSVQRFIEQAAQD  377
             D L Y  F P     L   E      +F+ I ++D+ VHHP++SFA  V++FI QAA D
Sbjct  298  LDQLRYVPFEPKLPSELVNHEGE---SLFSVIGKQDLFVHHPFESFAV-VEQFIAQAATD  353

Query  378  PQVLAIKQTLYRTSGDSPIIDALVDAAEAGKQVLAVVEIKARFDEQANITWARLLERAGV  437
            P VLAIKQTLYR SGDSPII +L+ AA+ GKQV  ++E+KARFDE+ NIT AR LE+AG
Sbjct  354  PDVLAIKQTLYRVSGDSPIIASLIKAADNGKQVTVLMEVKARFDEEHNITMARRLEKAGC  413

Query  438  HVVYGMVGLKTHCKLAMVIRDEGEGLRRYAHIGTGNYNPKTARQYEDLGLLTSNPIITED  497
            HV+YG+ GLKTH K+ MV+R E  G+RRY H+ TGNYN KTAR Y D G+ T N    +D
Sbjct  414  HVIYGLKGLKTHSKITMVVRREDAGIRRYVHLATGNYNGKTARMYTDCGIFTCNDEYGDD  473

Query  498  VARLFNHLSGMTAEKRYRRLLVAPESIRSGIIDAIEREIDNKKAGLPAGVRIKVNSIVDE  557
             +R FN +SG +    + + +VAP ++R  I++ I+REI+  K G  A +  K+NS++D+
Sbjct  474  ASRFFNLISGYSDPPIWNKFIVAPLNLREKIMELIDREIEFAKNGEEAYIIGKMNSLLDK  533

Query  558  RVIDALYRASRAGVPVDLWVRGICSIRPGVPGLSENIRVISILGRFLEHSRIFWFANGGR  617
             VI  LY AS AGV +DL  RGIC++RPG+ G+S+NI V SI+GRFLEH R+F+F NGG
Sbjct  534  EVIAKLYEASAAGVRIDLIGRGICTLRPGITGVSDNITVRSIVGRFLEHHRLFYFRNGGN  593

Query  618  PMVAIGSADLMHRNLDRRVEALVGLSNKQHVAEVE  652
              + + SAD M RNL+ RVE ++ + +K+H   V+
Sbjct  594  ESLYLSSADWMPRNLNERVELMIPIEDKRHKERVK  628
```

Figure S3: NCBI BLASTP results for aligning the query sequence pac:PPA0343 (KEGG orthology: K00973) from the *P. acnes* genome against the best contig assembled by GRASPx.
